# Coupling Chiral Cuboids with Wholly Auxetic Response

**DOI:** 10.34133/research.0463

**Published:** 2024-08-30

**Authors:** Jiajun Wang, Zhaochang Chen, Pengcheng Jiao, Amir H. Alavi

**Affiliations:** ^1^ Ocean College, Zhejiang University, Zhoushan, Zhejiang, China.; ^2^ Engineering Research Center of Oceanic Sensing Technology and Equipment, Ministry of Education, Hangzhou, Zhejiang, China.; ^3^ Hainan Institute, Zhejiang University, Sanya, Hainan, China.; ^4^ Department of Civil and Environmental Engineering, University of Pittsburgh, Pittsburgh, PA, USA.; ^5^ Department of Mechanical Engineering and Materials Science, University of Pittsburgh, Pittsburgh, PA, USA.

## Abstract

Auxetic materials have been extensively studied for their design, fabrication and mechanical properties. These material systems exhibit unique mechanical characteristics such as high impact resistance, shear strength, and energy absorption capacity. Most existing auxetic materials are two-dimensional (2D) and demonstrate half-auxetic behavior, characterized by a negative Poisson’s ratio when subjected to either tensile or compressive forces. Here, we present novel three-dimensional (3D) auxetic mechanical metamaterials, termed coupling chiral cuboids, capable of achieving negative Poisson’s ratio under both tension and compression. We perform experiments, theoretical analysis, and numerical simulations to validate the wholly auxetic response of the proposed coupling chiral cuboids. Parametric studies are carried out to investigate the effects of structural parameters on the elastic modulus and Poisson’s ratio of the coupling chiral cuboids. The results imply that the Poisson’s ratio sign-switching from negative to positive can be implemented by manipulating the thickness of Z-shaped ligaments. Finally, the potential application of the coupling chiral cuboids as inner cores for impact-resistant sandwich panels is envisioned and validated. Test results demonstrate a remarkable 49.3% enhancement in energy absorption compared to conventional solid materials.

## Introduction

The negative Poisson’s ratio (NPR) performance of auxetic materials and structures generally endows them with enhanced properties. These include improved energy absorption, fracture resistance, and shear resistance, distinguishing them from conventional materials with positive Poisson’s ratios [1–3]. The promising properties of auxetic structures and materials have opened up broad avenues for applications across various engineering and medical fields, including protective devices [4], morphing airfoils [5], and intravascular stents [6]. Natural auxetic materials and structures have been discovered by scientists, such as the observation of auxetic response in the nuclei of mouse embryonic stem cells during metastable transition states [7] and the demonstration of auxetic response in semi-fluorinated graphene induced by the chemical functionality of fluorine atoms [8]. However, natural auxetic materials are scarce and difficult to control, leading to a predominant reliance on artificially designed and fabricated auxetic materials and structures [9,10]. Enhancing the energy absorption properties of auxetic materials and structures has been a major research focus in recent years [3]. 

Chiral mechanical metamaterials constitute a specific subclass of auxetic materials. The evolution of chiral mechanical metamaterials has followed a developmental trajectory from 2D to 3D structures [1,11,12]. Chiral honeycomb plate capable of maintaining a Poisson’s ratio of −1 across a wide deformation range marks the advent of chiral mechanical metamaterials [13]. Subsequently, a series of 2D chiral mechanical metamaterials were designed and developed, including auxetic meta-tetrachiral structures, cropped rotational-polygon structures, double-negative mechanical metamaterials, and hierarchical anti-tetrachiral metastructures [14–17]. Inspired by these advancements, researchers extended their designs into the 3D realm by periodically expanding chiral units in 3D space [18–21]. This led to the creation of 3D auxetic chiral isotropic lattices featuring rigid cubical cells and deformable ligaments [22]. Innovative 3D twist chiral mechanical metamaterials were developed to overcome design limitations associated with coordinate transformations and applications relying on mode conversion [23]. Additionally, 3D chiral tubes with hexagonal cells and Z-shaped ligaments were reported, achieved through the rolling of 2D chiral plates [24,25]. This ongoing evolution from 2D to 3D structures underscores the versatility and potential of chiral mechanical metamaterials in various engineering applications.

At the current stage, the majority of auxetic materials and structures are 2D systems and exhibit the half-auxetic response [26–32]. Achieving an NPR under both tension and compression in auxetic materials can potentially unlock a range of unique mechanical properties for various applications. To this end, we introduce coupling chiral cuboids, a novel class of 3D chiral mechanical metamaterials exhibiting wholly auxetic behavior under both tension and compression. The auxetic behavior of these structures is comprehensively investigated through experiments, theoretical modeling, and numerical simulations. We conduct parametric studies to understand the effects of structural parameters on the elastic modulus and Poisson’s ratio of the coupling chiral cuboids. The Poisson’s ratio sign-switching from negative to positive is implemented by manipulating the thickness of the designed Z-shaped ligaments. Finally, we showcase the potential application of proposed chiral cuboids as the inner cores of impact-resistant sandwich panels.

## Results and Discussion

### Structural design and mechanical analysis

**Fig. 1.**
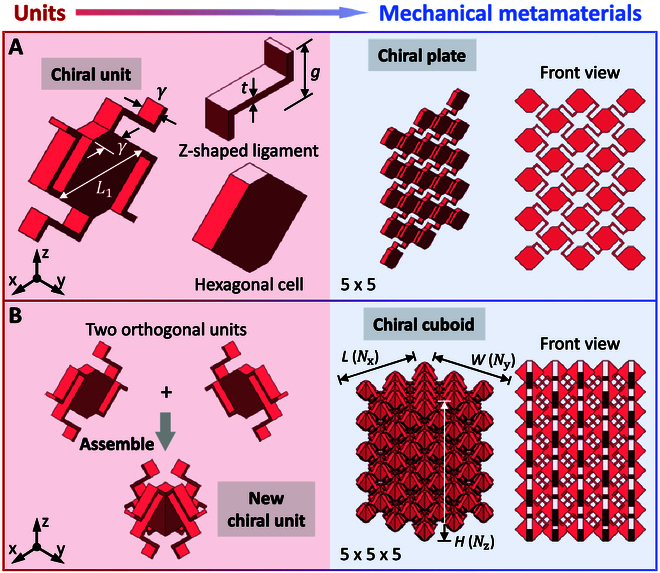
Structural design of coupling chiral cuboids from units to mechanical metamaterials. (A) Chiral unit composed of a hexagonal cell and 4 Z-shaped ligaments for constituting the chiral plates. (B) New chiral unit assembled by 2 orthogonal units of chiral plates for constituting the chiral cuboids.

Developing chiral cuboids with a wholly auxetic response is essential for several reasons. These 3D auxetic structures can achieve an NPR under both tension and compression. Thus, they are capable of responding effectively to both tensile and compressive loading conditions. They expand in both directions when stretched and uniformly densify under compression. This dual-response capability holds the potential for designing structures that can withstand a broader range of mechanical stresses. Moreover, such coupling chiral cuboids could theoretically demonstrate improved energy absorption and dissipation properties compared to materials exhibiting only one-sided auxetic behavior. These characteristics can pave the way for innovative solutions in applications where impact resistance and shock absorption are crucial, such as protective gear and structural reinforcements, or in scenarios where a more natural stress response is desired, like in biomedical implants. Figure 1 illustrates the structural design of the proposed coupling chiral cuboids from units to mechanical metamaterials. Figure 1A presents a coupling chiral unit composed of a hexagonal cell and 4 Z-shaped ligaments. The hexagonal cell is obtained by cutting the square with the diagonal length of *L*_1_, and the length of cut portions is equal to the thickness of chiral unit *γ*. The effective length and thickness of Z-shaped ligaments are *g* and *t*. The chiral plates are formed by periodically expanding the chiral units that are connected by Z-shaped ligaments. Figure 1A demonstrates a 5 × 5 chiral plate. The chiral plates are verified with the wholly auxetic response by numerical simulations (Note S1). In order to constitute the chiral cuboids, the new chiral unit is designed by assembling 2 orthogonal units of chiral plates (Fig. 1B). The number of units of chiral cuboids in the *x*, *y*, and *z* directions are *N*_x_, *N*_y_, and *N*_z_, and the length (*x* direction), width (*y* direction), and height (*z* direction) are *L*, *W*, and *H*, respectively. A 5 × 5 × 5 coupling chiral cuboid is displayed in Fig. 1B.

**Fig. 2.**
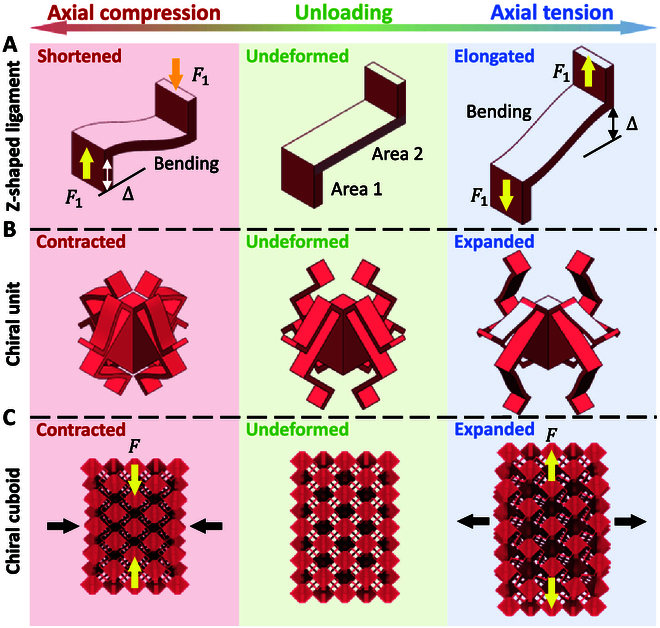
Deformation analysis for explaining the formation mechanism of wholly auxetic response of coupling chiral cuboids. (A) Z-shaped ligament. (B) Chiral unit. (C) Assembled metamaterial structure.

Figure 2 presents the structural deformations of Z-shaped ligament, coupling chiral unit, and assembled metamaterial structure under axial tension and compression. According to the analysis, the wholly auxetic response of chiral cuboids mainly stems from the bending of area 2 of Z-shaped ligaments. In particular, the bending will result in the elongation (shortening) of Z-shaped ligaments under axial tension (compression) (Fig. 2A), which leads to the lateral expansion (contraction) of chiral units and overall structure (Fig. 1B and C). Theoretical modeling of elastic modulus and Poisson’s ratio of chiral cuboids are conducted to quantitatively characterize the mechanical response in Methods. In summary, to address the challenges of auxetic materials and structures in achieving 3D systems and a wholly auxetic response, we propose 2 innovative structural design strategies. First, the ligaments are designed in a Z shape, enabling the assembled metamaterial structures to achieve a wholly auxetic response through their properties of elongation and shortening under tension and compression, respectively. Second, the 3D units of coupling chiral cuboids are constructed by assembling 2 orthogonal 2D units of chiral plates, thereby achieving a breakthrough from 2D systems (chiral plates) to 3D systems (coupling chiral cuboids).

### Experiments and numerical simulations

**Fig. 3.**
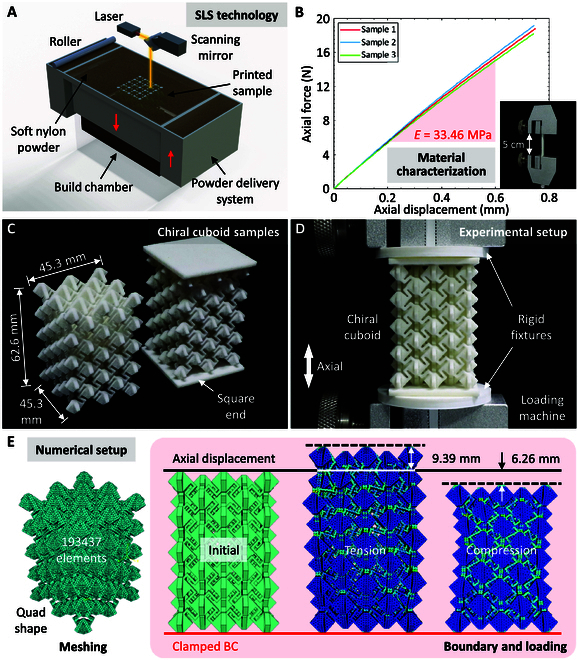
Experimental and numerical setup. (A) Fabrication diagram of the coupling chiral cuboid samples using the SLS technology. (B) Tensile tests of soft nylon dumbbell samples for the material characterization. (C) Chiral cuboid samples. (D) Experimental setup of axially tensile and compressive tests for the chiral cuboid sample. (E) Meshing, boundary, and loading conditions of chiral cuboids under axial tension and compression in numerical simulations.

Experiments and numerical simulations are conducted to validate the wholly auxetic response of coupling chiral cuboids. The results are compared with the theoretical models. A parametric analysis is subsequently performed. Figure 3A presents the fabrication principle of the coupling chiral cuboid samples using selective laser sintering (SLS) technology. Soft nylon is used for printing the samples. The Young’s modulus (*E*) of soft nylon material is determined as 33.46 MPa by the tensile tests of soft nylon dumbbell samples (Fig. 3B). The structural parameters of soft nylon dumbbell samples and experimental setup of tensile tests are summarized in Note S2. The material Poisson’s ratio is taken as 0.4 (i.e., the Poisson’s ratio of nylon), and the material density is measured as 1.11 g/cm^3^. Figure 3C demonstrates the fabricated chiral cuboid samples with the structural parameters of *γ* = 2mm, *L*_1_ = 10mm, *g* = 4mm, *t* = 1mm, *N*_x_ = 5, *N*_y_ = 5, *N*_z_ = 5, *L* = 45.3mm, *W* = 45.3mm, and *H* = 62.6mm. Figure 3D displays the experimental setup of axially tensile and compressive tests of chiral cuboid samples. The square ends are bonded to the 3D-printed polylactic acid (PLA) rigid fixtures using the instant-drying adhesive to ensure the uniform distribution and stability of applied axial displacement. The instruments and operating procedures for preparation and mechanical tests are detailed in Methods.

The tensile and compressive response of coupling chiral cuboids with different *t* are numerically simulated in Abaqus/CAE using the static/general solving algorithm. The structural and material parameters used in numerical simulations are summarized in Note S6. It is worthwhile pointing out that *t* is the uniquely tunable structural parameter when the overall dimensions of chiral cuboids (i.e., *L*, *W*, and *H*) are fixed. Figure 3E presents the meshing, boundary, and loading conditions. In numerical simulations, the bottom parts are clamped, and the upper parts are subjected to an axial displacement. In particular, the displacement is set as 9.39 mm (i.e., axial strain is 15%) under axial tension and 6.26 mm (i.e., axial strain is 10%) under axial compression. For the chiral cuboids with different *t*, the models are meshed into 193,437 (*t* = 0.5mm), 226,225 (*t* = 1mm), 214,198 (*t* = 1.5mm), 247,896 (*t* = 2mm), and 265,492 elements (*t* = 2.5mm) with Tet shape and Free technique, respectively.

### Wholly auxetic response

**Fig. 4.**
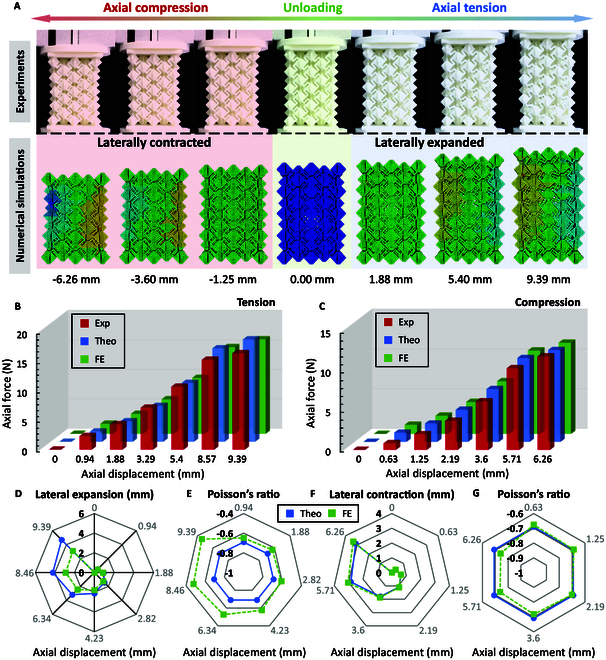
Comparisons between theoretical, experimental, and numerical results. (A) Comparison of deformed configurations of chiral cuboid between experiments and numerical simulations. Comparisons of axial force–axial displacement relationship between experiments, theoretical analysis, and numerical simulations under (B) axial tension and (C) compression. Comparisons of (D) lateral expansion–axial displacement and (E) Poisson’s ratio–axial displacement relationships between theoretical analysis and numerical simulations under axial tension. Comparisons of (F) lateral contraction–axial displacement and (G) Poisson’s ratio–axial displacement relationships between theoretical analysis and numerical simulations under axial compression.

Figure 4 compares the results of experiments, theoretical analysis, and numerical simulations. Figure 4A presents the deformed configurations of the fabricated coupling chiral cuboid with the structural parameters of *γ* = 2mm, *L*_1_ =10mm, *g* = 4mm, *t* = 1mm, *N*_x_ = 5, *N*_y_ = 5, and *N*_z_ = 5 under axial compression and tension in experiments and numerical simulations. The chiral cuboid exhibits a fully auxetic response, meaning it expands laterally under axial tension and contracts laterally under axial compression, as observed in both experiments and numerical simulations. In order to more prominently observe the wholly auxetic response of chiral cuboid, the deformation process of chiral cuboid under axial tension and compression in numerical simulations is displayed, and the deformation scale factor is set as 2 in the lateral deformation directions (Note S3).

Figure 4B and C compares the axial force–axial displacement relationship between experiments, theoretical analysis, and numerical simulations under axial tension and compression. Figure 4D and F uses radar charts to compare the relationships between lateral expansion/axial displacement and lateral contraction/axial displacement from theoretical analysis and numerical simulations under axial tension and compression. Based on these results (Fig. 4D and F), comparisons of Poisson’s ratio as it varies with axial displacement are presented for both theoretical analysis and numerical simulations under axial tension and compression (Fig. 4E and G). It is important to note that the lateral deformation of chiral cuboids under axial loads is not uniform along the axial direction, leading to different Poisson’s ratios at different axial positions. Therefore, the Poisson’s ratio reported in this study is an equivalent Poisson’s ratio determined from the maximum lateral deformation. Detailed explanations of the calculation and measurement methods for this equivalent Poisson’s ratio can be found in Note S4. Overall, the theoretical analysis, experiments, and numerical simulations demonstrate acceptable agreement in their results. The probable sources of error include the following: In experiments, the material (soft nylon) shows nonlinearity with increasing loading displacement. However, both theoretical analysis and numerical simulations assume linear elasticity for the material (Fig. 4B). In numerical simulations, the clamped ends restrict lateral deformation as loading displacement increases, a factor not accounted for in the theoretical analysis (Fig. 4D).

### Poisson’s ratio sign-switching

**Fig. 5.**
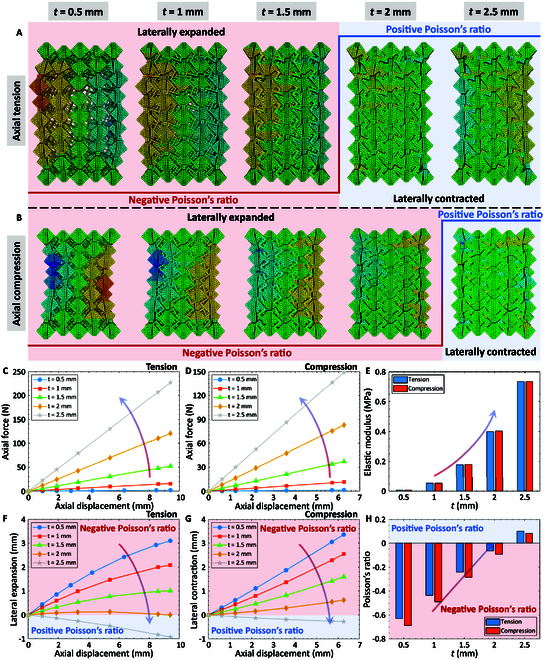
Poisson’s ratio sign-switching of coupling chiral cuboids by manipulating the thickness of Z-shaped ligaments *t*. Deformed configurations of chiral cuboids with different *t* under (A) axial tension and (B) compression. Effects of *t* on the axial force–axial displacement relationship of chiral cuboids under (C) axial tension and (D) compression. Influences of *t* on the lateral deformation–axial displacement relationship of chiral cuboids under (F) axial tension and (G) compression. Variations of (E) elastic modulus and (H) Poisson’s ratio of chiral cuboids with *t.*

The coupling chiral cuboids are validated with the structure-induced Poisson’s ratio sign-switching via numerical simulations. The chiral units and overall structures of chiral cuboids with different Z-shaped ligaments *t* are displayed in Note S6. Figure 5A and B compares the deformed configurations of chiral cuboids with different *t* under axial tension and compression, respectively. In order to more prominently demonstrate the Poisson’s ratio characteristics of chiral cuboids, the deformation scale factor is set as 2 in the lateral deformation directions. The Poisson’s ratio of the chiral cuboids exhibits a sign-switch from negative to positive as *t* increases. Figure 5C to H quantitatively investigates the influences of *t* on the elastic modulus and Poisson’s ratio of chiral cuboids. Figure 5C and F presents the axial force–axial displacement and lateral expansion–axial displacement relationships of chiral cuboids with different *t* under axial tension, while Fig. 5D and G demonstrates the axial force–axial displacement and lateral contraction–axial displacement relationships of chiral cuboids with different *t* under axial compression. The elastic modulus and Poisson’s ratio of chiral cuboids with different *t* are calculated by the results of time step 2 in Fig. 5C, D, F, and G, as shown in Fig. 5E and H. The results indicate that the elastic modulus and Poisson’s ratio of chiral cuboids under axial tension and compression are positively correlated with *t*. In addition, the tensile and compressive elastic modulus of chiral cuboids are practically equal, while the tensile Poisson’s ratio of chiral cuboids is larger than the compressive Poisson’s ratio. The sign-switching of Poisson’s ratio in chiral cuboids is attributed to the varying ratio of thickness to width (*t*/*w*) in Z-shaped ligaments. Specifically, as *t* and *t*/*w* increase, the bending deformation within area 2 of the Z-shaped ligaments diminishes, while shear deformation becomes increasingly prevalent. Consequently, the chiral units lose their characteristic expansion under axial tension and contraction under axial compression, causing the Poisson’s ratio of the chiral cuboids to transition from negative to positive.

### Application as the impact-resistant sandwich panels

**Fig. 6.**
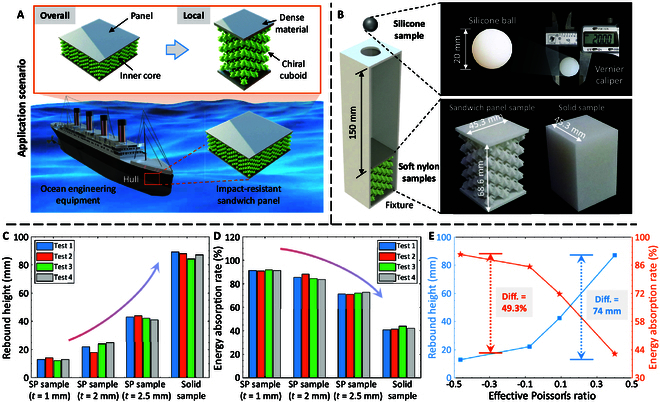
Potential application of the proposed coupling chiral cuboids as the impact-resistant sandwich panels. (A) Chiral cuboids serve as the inner cores of impact-resistant sandwich panels to develop the overlays to protect the ocean engineering equipment. (B) Experimental setup of ball rebounding tests that validate the impact-resistant performance of sandwich panels based on the chiral cuboids. Comparisons of (C) rebound height and (D) energy absorption rate between sandwich panel and normal samples. (E) Effects of effective Poisson’s ratio on the average rebound height and energy absorption rate.

The proposed coupling chiral cuboids can serve as the inner cores of impact-resistant sandwich panels to develop the overlays for the ocean engineering equipment (Fig. 6A). The ball rebounding tests are conducted to verify the impact-resistant performance of sandwich panels based on the chiral cuboids and deeply study the effect of Poisson’s ratio on the impact energy absorption. Figure 6B presents the experimental setup of ball rebounding tests; in particular, the sandwich panel samples with different thickness of Z-shaped ligaments *t* (1, 2, and 2.5 mm) and solid sample with the identical dimensions are respectively placed at the bottom of 3D-printed PLA fixture, and a silicone ball with a diameter of 20 mm is dropped from the round hole at the top of fixture (the drop height is 150 mm). The sandwich panel and solid samples are fabricated by the SLS technology using soft nylon. Figure 6C and D compares the rebound height and energy absorption rate between sandwich panel and solid samples. It can be seen that the sandwich panel samples demonstrate a smaller rebound height and a larger energy absorption rate than the solid sample, which certifies that the sandwich panels with the inner cores of chiral cuboids possess superior impact-resistant performance than the solid materials. Additionally, the rebound height and energy absorption rate are positively and negatively correlated with *t*, respectively. The influences of effective Poisson’s ratio on the average rebound height and energy absorption rate are obtained from the results of Fig. 6C and D (Fig. 6E). The effective Poisson’s ratios of sandwich panel and solid samples used in the ball rebounding tests are summarized in Note S7. The results reveal that the effective Poisson’s ratio proportionally affects the rebound height, while anti-proportionally influences the energy absorption. Moreover, compared with the solid sample, the sandwich panel sample can achieve a 49.3% growth in the energy absorption rate. It is worth pointing out that more durable and corrosion-resistant materials, such as aluminum alloys, titanium alloys, and fiber-reinforced composites, are necessary to fabricate sandwich panels based on chiral cuboids for engineering applications. The challenges posed by these materials may include (a) a lack of large-scale preparation technologies to manufacture the overlays as a single unit for the proposed sandwich panels and (b) preparing the local structures of sandwich panels separately and assembling them to form the overlays, which would significantly increase manufacturing time and cost.

## Conclusion

In this research, a new class of 3D chiral mechanical metamaterials (i.e., coupling chiral cuboids) is developed with wholly auxetic response under both tension and compression and Poisson’s ratio sign-switching. Experiments, theoretical analysis, and numerical simulations are conducted and compared to validate the wholly auxetic response of coupling chiral cuboids. The comparative results demonstrate satisfactory agreement. Parametric studies are carried out via numerical simulations to investigate the effects of structural parameters on the *E* and Poisson’s ratio of chiral cuboids. The results indicate that the Poisson’s ratio of chiral cuboids exhibits a sign-switch from negative to positive as the thickness of Z-shaped ligaments *t* increases. The potential application of chiral cuboids as the inner cores of impact-resistant sandwich panels is envisioned and validated. The impact-resistant performance of sandwich panels utilizing chiral cuboids is verified through ball rebounding tests. These tests reveal a 49.3% increase in the energy absorption rate for the chiral cuboid-enabled sandwich panel sample compared to the solid sample. Future research in this area can focus on multi-axial auxeticity. Exploring designs that achieve NPR in all directions could involve designing even more complex 3D geometries or incorporation of additional material properties. Moreover, wholly auxetic structures can be combined with other materials to create multifunctional composites with an array of combined properties. In this case, the dynamic mechanical behavior of such composite chiral cuboids should be comprehensively studied under various loading conditions, including dynamic impact, cyclic loading, and high strain rates.

## Methods

### Theoretical analysis

This section theoretically characterizes the elastic modulus (*E*_z_) and Poisson’s ratio (_v_) of coupling chiral cuboids. Assuming the chiral cuboids are subjected to an axial force and reach the equilibrium, the responsive force in area 1 of Z-shaped ligaments *F*_1_ is:

$$F_{1}=\frac{\sqrt{2}F}{2N_{x}N_{y}-N_{x}-N_{y}},$$ (1)

where *N*_x_ and *N*_y_ present the number of chiral units in the *x* and *y* directions, respectively. The free body diagram (FBD) that explains the relationship between *F* and *F*_1_ given in Eq. 1 is drawn in Note S8. Note that *F* takes a positive number under axial tension, while *F* is negative under axial compression. Area 2 of Z-shaped ligaments can be considered as the beams clamped at both ends so that the bending-induced deflection of Z-shaped ligaments can be expressed as:

$$∆=\frac{F_{1}}{12\mathrm{EI}}{\left[\frac{\left(L_{1}-\gamma \right)}{2\cos\left(\pi /4\right)}\right]}^{3}=\frac{F{\left(L_{1}-\gamma \right)}^{3}}{2E\gamma t^{3}\left(2N_{x}N_{y}-N_{x}-N_{y}\right)}$$ (2)

where $I=\frac{\gamma t^{3}}{12}$ denotes the cross-section moment of inertia of area 2 of Z-shaped ligaments. Integrating the deformation of Z-shaped ligaments, the lateral deformation in the *x* and *y* directions (*D*_x_ and *D*_y_), and axial deformation (*D*_z_) of chiral cuboids can be written as:

Dx=Nx−1∆cosπ/4Dy=Ny−1∆sinπ/4Dz=2Nz−1∆sinπ/4. (3)

Considering that the displacement control method is chosen in experiments and numerical simulations, we assume the axial displacement *D*_z_ as the independent variable. Substituting Eq. 2 into Eq. 3, *F* can be obtained as:

$$F=\frac{\sqrt{2}E\gamma t^{3}\left(2N_{x}N_{y}-N_{x}-N_{y}\right)}{{\left(L_{1}-\gamma \right)}^{3}\left(N_{z}-1\right)}D_{z}.$$ (4)

Applying *D*_z_ to express *D*_x_ and *D*_y_, we have

Dx=Nx−12Nz−1DzDy=Ny−12Nz−1Dz. (5)

Consequently, *E*_z_ and ν in the *x* and *y* directions (ν_x_ and ν_y_) can be calculated as:

Ez=FHDzLW=2Eγt3H2NxNy−Nx−NyL1−γ3LWNz−1νx=−DxHDzW=−Nx−1H2Nz−1Wνy=−DyHDzL=−Ny−1H2Nz−1W. (6)

Equation 6 reveals that the *E*_z_ and ν of chiral cuboids can be uniquely determined by the material and structural parameters. The developed theoretical models are solely applicable to the small-deformation conditions. In addition, the accuracy of theoretical models can be improved by reducing the thickness of Z-shaped ligaments *t* or enlarging the number of chiral units. The influence of *t* on the effectiveness of theoretical models is discussed in Note S9.

### Fabrication and mechanical tests

The coupling chiral cuboid and dumbbell samples in experiments are fabricated using soft nylon material. The samples were printed using an industrial-grade SLS printer (i.e., HT403P, FARSOON Technologies Co. Ltd., Changsha, China). The 3D printer uses a laser to fuse the soft nylon powder into the samples. In particular, the laser traces the pattern of each cross-section of sample models and replicates it onto the powder bed. Once one layer is built, the build chamber is lowered and construction of new layer begins on the top of previous layer. The PLA fixtures are prepared using fused deposition modeling (FDM) technology. A dual-extruder 3D printer (i.e., Rasie3D Pro2) is used for this purpose. The platform temperature, printing temperature, filling speed, and filling rate are separately set as 60 °C, 205 °C, 60 mm/s, and 50% during the printing. For the mechanical tests, a computerized electronic fatigue testing machine (manufactured by the Han Shen Automation Co. Ltd., Jinan, China) was used to apply the axial loads in the displacement control mode. The loading speed is set as 20 and 5 mm/min for the mechanical tests of soft nylon chiral cuboid and dumbbell samples, respectively.

## Data Availability

The data that support the findings of this study are available from the corresponding author upon reasonable request.

## Supplementary Materials

Supplementary 1 Notes S1 to S9 Figs. S1 to S9 Tables S1 to S3 Movies S1 to S4
