# Supplementary material for: Coupling Chiral Cuboids with Wholly Auxetic Response
Source: Research (Wash D C). 2024 Aug 30;7:0463. doi: 10.34133/research.0463 (PMC11362674; doi:10.34133/research.0463)
Supplement: Supplementary 1 — Notes S1 to S9 Figs. S1 to S9 Tables S1 to S3 Movies S1 to S4 [file research.0463.f1.zip › Revised Supplementary Information.docx]

**Supplementary Information for**

**Coupling Chiral Cuboids with Wholly Auxetic Response**

**Jiajun Wang^a^, Zhaochang Chen^a^, Pengcheng Jiao^a,b,c,1^, Amir H. Alavi^d,e,^^[[1]](#footnote-1)^**

*^a^Ocean College, Zhejiang University, Zhoushan, Zhejiang, China*

*^b^Engineering Research Center of Oceanic Sensing Technology and Equipment, Ministry of Education, Hangzhou, Zhejiang, China*

*^c^Hainan Institute, Zhejiang University, Sanya, Hainan, China*

*^d^Department of Civil and Environmental Engineering, University of Pittsburgh, Pittsburgh, PA, USA*

*^e^Department of Mechanical Engineering and Materials Science, University of Pittsburgh, Pittsburgh, PA, USA*

# Supplementary Note 1: Wholly auxetic response of chiral plates

We numerically characterize the chiral plate with the structural parameters of $\gamma=2 mm$, $L_{1}=10 \mathrm{mm}$, $g=4 \mathrm{mm}$, $t=0.5 \mathrm{mm}$, $N_{x}=5$ and $N_{y}=5$ in Abaqus/CAE using the static/general solving algorithm. The material is chosen as the soft nylon, whose material parameters are provided in Results and Discussion. The chiral plate is observed with the wholly auxetic response (Fig. S1A). Fig. S1B presents the variations of lateral deformation and Poisson’s ratio of chiral plate with the axial displacement.


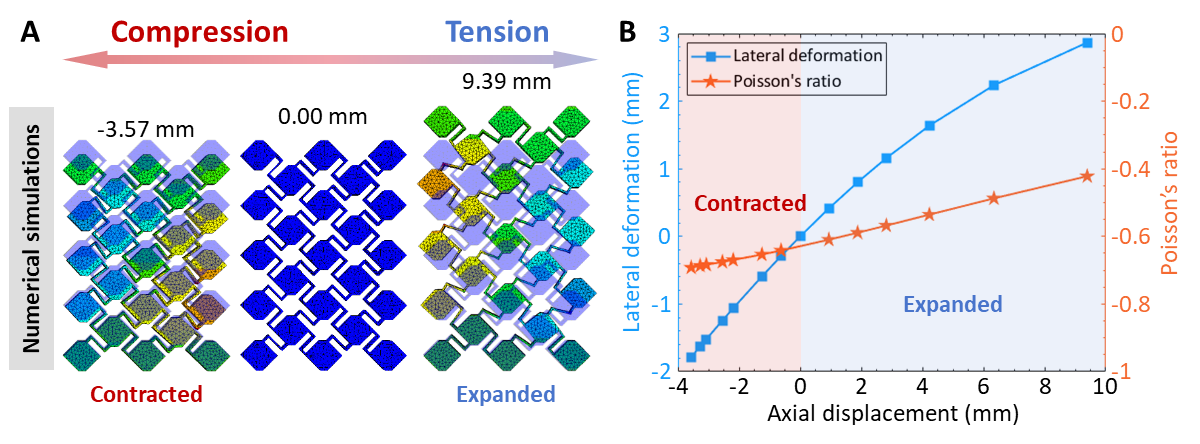


**Fig. S1.** Characterization of chiral plates via numerical simulations. (A) Wholly auxetic response of chiral plate in numerical simulations. (B) Variations of lateral deformation and Poisson’s ratio of chiral plate with the axial displacement.

# Supplementary Note 2: Tensile tests of soft nylon dumbbell samples

The tensile tests of soft nylon dumbbell samples were conducted to calibrate the Young’s modulus of material for the experimental and numerical analysis of chiral cuboids. Figs. S2A and S2B present the geometric dimensions and test samples of soft nylon dumbbell samples, respectively. Fig. S2C demonstrates the experimental setup of tensile tests. Three samples were separately tested once to obtain three sets of force-displacement relationships. The loading displacement (strain) was controlled as 10 mm (20%). Table S1 summaries the geometric properties of soft nylon dumbbell samples and loading conditions in the tensile tests.


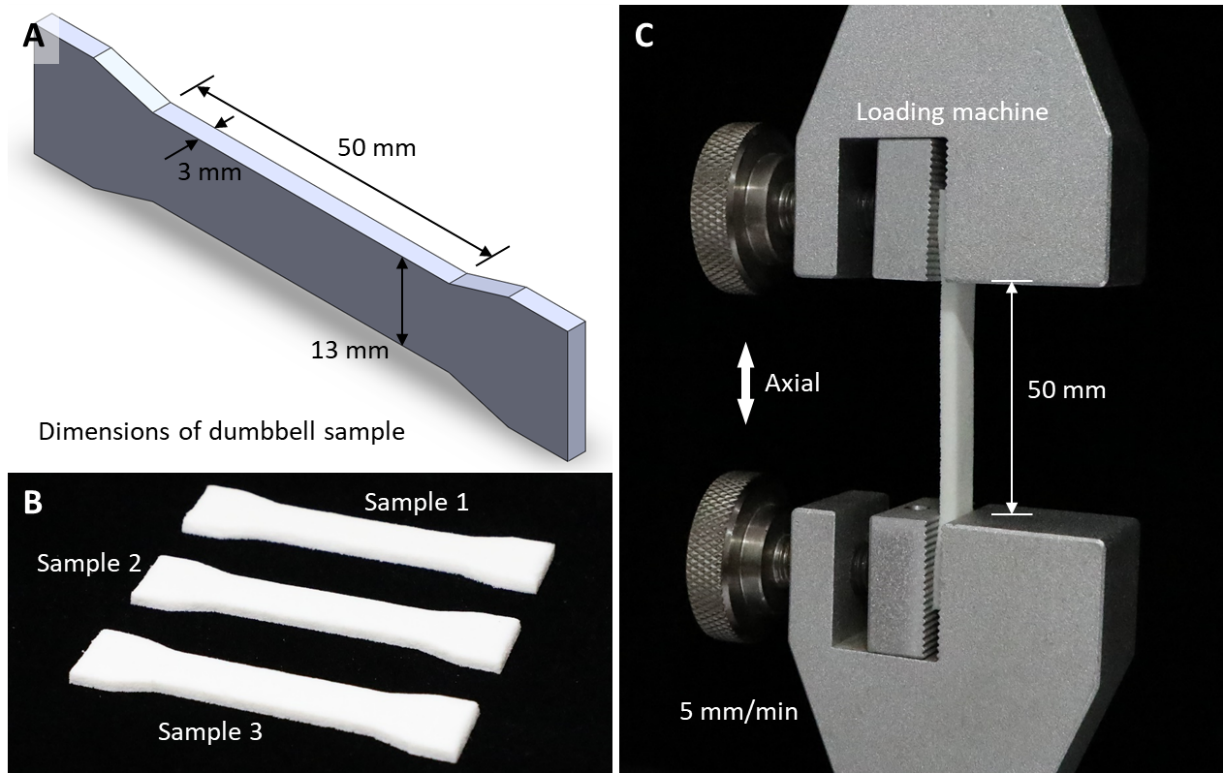


**Fig. S2.** Geometric dimensions and tensile tests of soft nylon dumbbell samples. (A) Geometric dimensions of soft nylon dumbbell samples. (B) Test samples of soft nylon dumbbell samples fabricated by SLS technology. (C) Experimental setup of tensile tests of soft nylon dumbbell samples.

**Table. S1.** Geometric properties of soft nylon dumbbell samples and loading conditions in the tensile tests.

| Sample | Thickness (mm) | Length (mm) | Width (mm) | Loading type | Loading speed (mm/min) |
| --- | --- | --- | --- | --- | --- |
| 1 | 3 | 50 | 13 | Displacement control | 5 |
| 2 |  |  |  |  |  |
| 3 |  |  |  |  |  |

# Supplementary Note 3: Wholly auxetic response of coupling chiral cuboids

Figs. S3A and S3B present the deformation process of coupling chiral cuboid with the structural parameters of $\gamma=2 mm$, $L_{1}=10 \mathrm{mm}$, $g=4 \mathrm{mm}$, $t=1 \mathrm{mm}$, $N_{x}=5$, $N_{y}=5$ and $N_{z}=5$ under axial tension and compression in numerical simulations, and the wholly auxetic response is observed. In order to more prominently demonstrate the negative Poisson’s ratio characteristic of chiral cuboid, the deformation scale factor is set as 2 in the lateral deformation directions.


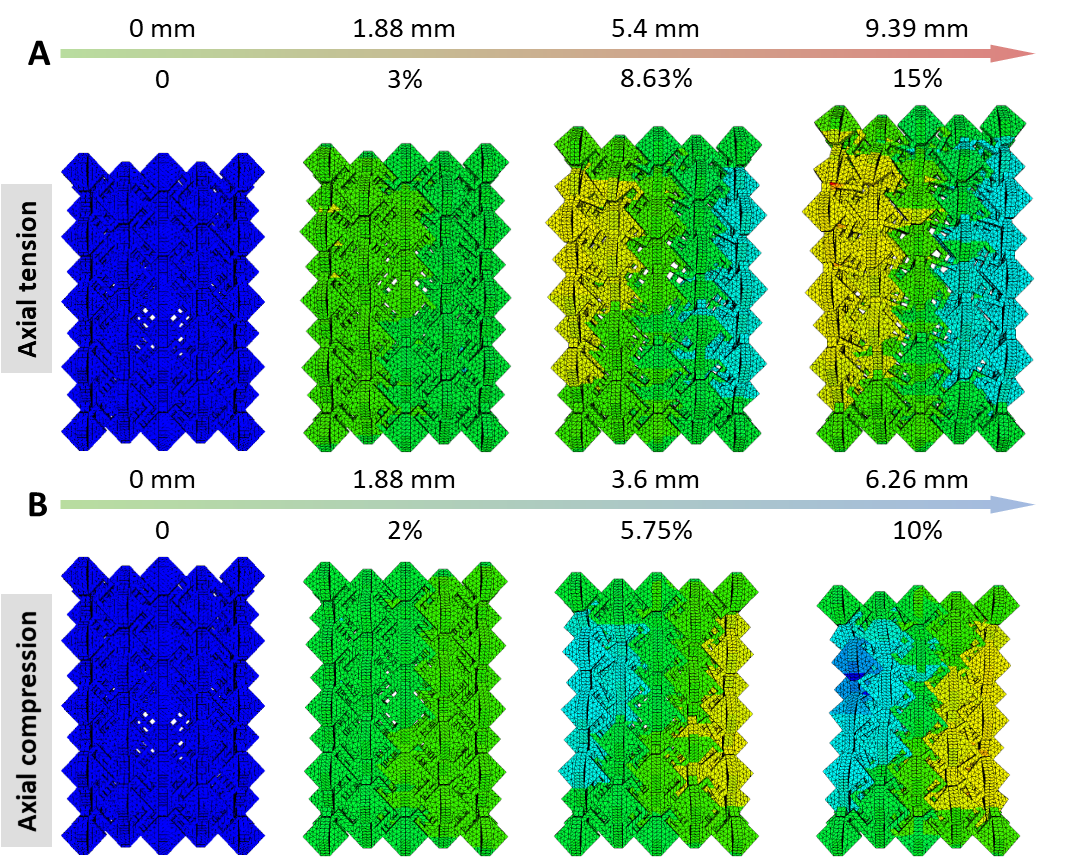


**Fig. S3.** Deformation process of coupling chiral cuboid under (A) axial tension and (B) compression.

# Supplementary Note 4: Calculation and measurement methods of equivalent Poisson’s ratio of coupling chiral cuboids

The lateral deformation of coupling chiral cuboids under axial loads is axially different, which results in different Poisson’s ratios calculated or measured at different axial positions. Thus, the Poisson’s ratio of chiral cuboids in this research is an equivalent Poisson’s ratio, whose value is obtained from the maximum lateral deformation. Fig. S4 introduces the calculation and measurement methods of equivalent Poisson’s ratio of chiral cuboids using the case of Poisson’s ratio in the $y$ direction. In particular, the positions where the maximum lateral deformation occurs are marked with two blue dots in the figure. The maximum lateral deformation of chiral cuboids $D_{y}$ is obtained by calculating or measuring the lateral displacement between the blue dots ($W_{\mathrm{comp}}$ and $W_{\mathrm{ten}}$), and subtracting the width of chiral cuboids $W$. Substituting the obtained $D_{y}$ into Eq. (6), the equivalent Poisson’s ratio of chiral cuboids $\nu_{y}$ is determined.


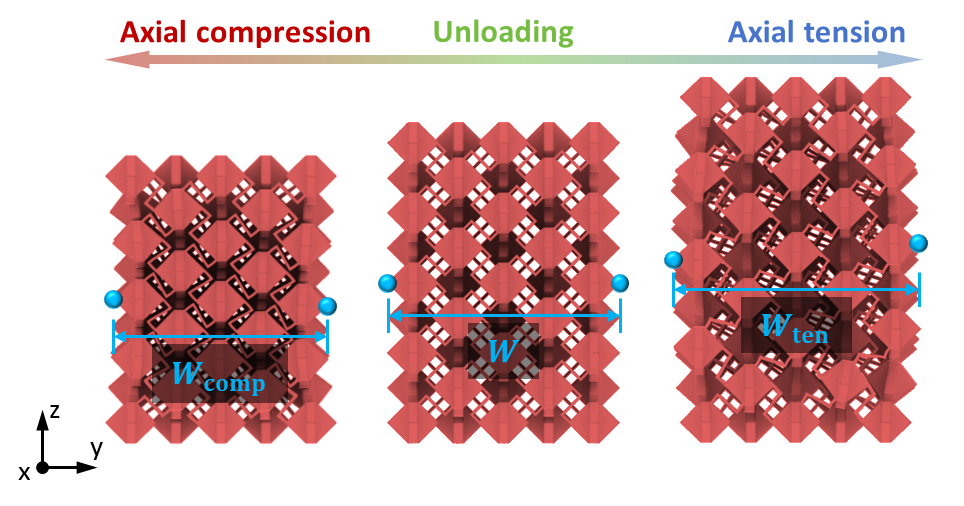


**Fig. S4.** Calculation and measurement methods of equivalent Poisson’s ratio of coupling chiral cuboids.

# Supplementary Note 5: Comparison of force-displacement curves

This section presents two-dimensional curve charts to compare the axial force-axial displacement relationship between experiments, theoretical analysis and numerical simulations to demonstrate the consistency throughout the entire compression and tension processes, as presented in Fig. S5.


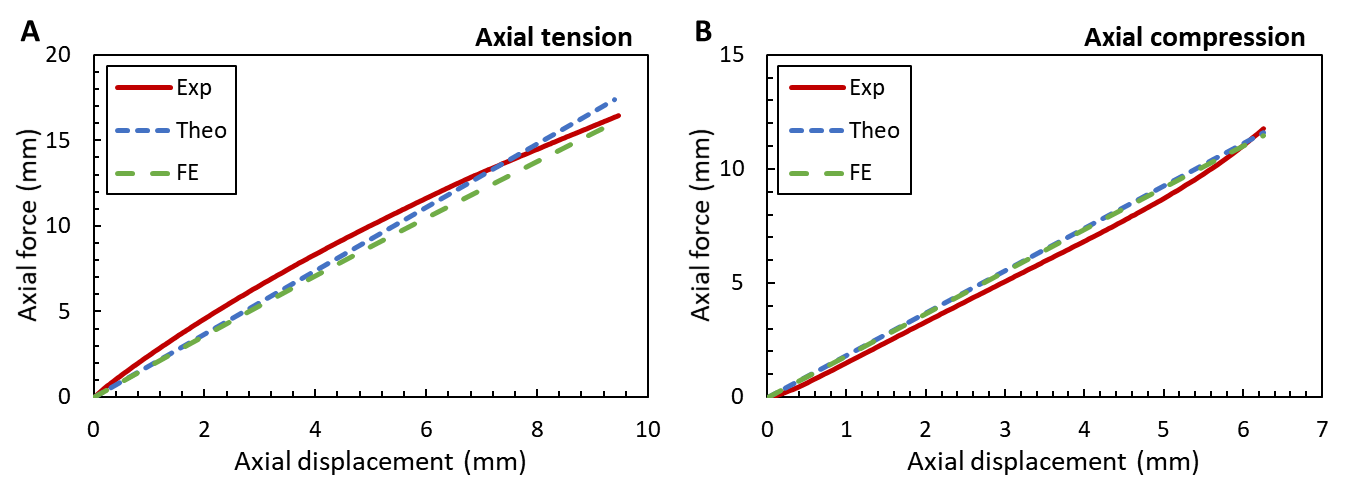


**Fig. S5.** Comparisons of axial force-axial displacement relationship between experiments, theoretical analysis and numerical simulations under (A) axial tension and (B) compression.

# Supplementary Note 6: Coupling chiral cuboids with different thickness of Z-shaped ligaments $\boldsymbol{t}$

Parametric studies are carried out via numerical simulations to investigate the effects of thickness of Z-shaped ligaments $t$ on the elastic modulus and Poisson’s ratio of coupling chiral cuboids. Figs. S6A and S6B present the chiral units and overall structures of chiral cuboids with different $t$, respectively. Table S2 summarizes the structural and material parameters used in numerical simulations.


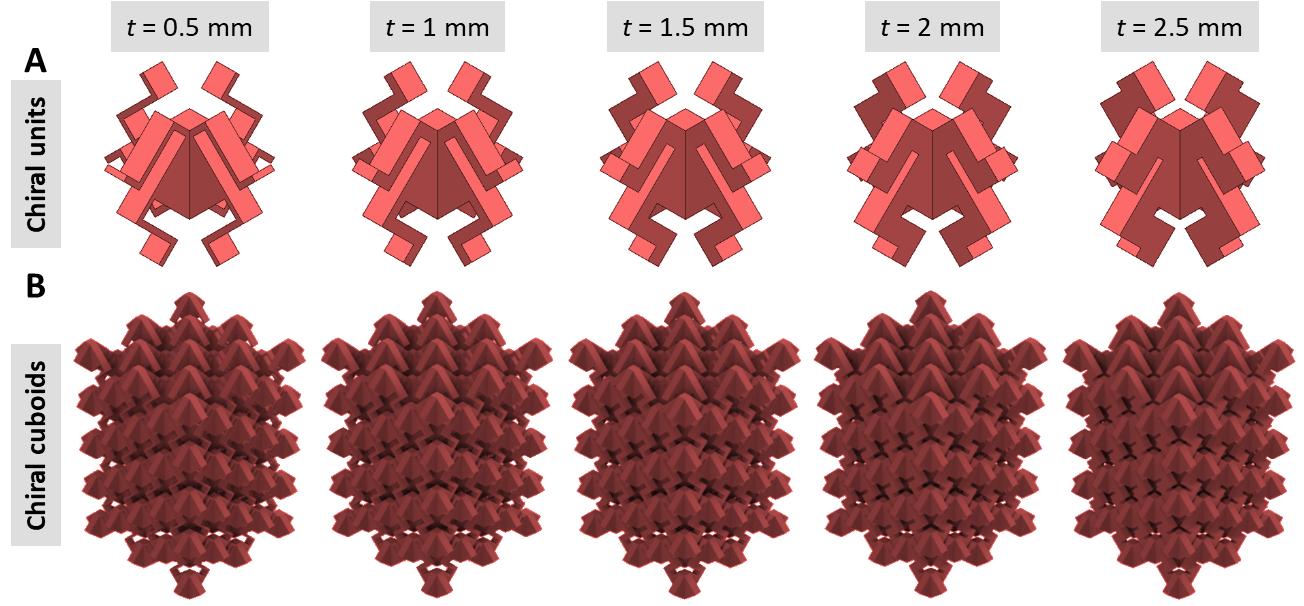


**Fig. S6.** (A) Chiral units and (B) overall structures of chiral cuboids with different thickness of Z-shaped ligaments $t$.

**Table. S2.** Structural and material parameters of chiral cuboids in numerical simulations.

| **Structural parameters** | $\gamma$ (mm) | $L_{1}$ (mm) | $g$ (mm) | | $t$ (mm) | $N_{x}$ (mm) | | $N_{y}$ (mm) | $N_{z}$ (mm) |
| --- | --- | --- | --- | --- | --- | --- | --- | --- | --- |
|  | 2 | 10 | 4 | | 0.5 | 5 | | 5 | 5 |
|  |  |  |  |  | 1 |  |  |  |  |
|  |  |  |  |  | 1.5 |  |  |  |  |
|  |  |  |  |  | 2 |  |  |  |  |
|  |  |  |  |  | 2.5 |  |  |  |  |
| **Material parameters** | Density (g/cm^3^) | | | Young’s modulus (MPa) | | | Poisson’s ratio | | |
|  | 1.11 | | | 33.46 | | | 0.4 | | |

# Supplementary Note 7: Effective Poisson’s ratio of sandwich panel and solid samples

In order to verify the impact-resistant performance of sandwich panels with the inner cores of coupling chiral cuboids and deeply investigate the effect of Poisson’s ratio on the impact energy absorption, sandwich panel samples with different thickness of Z-shaped ligaments $t$ (1 mm, 2 mm and 2.5 mm) and solid sample were fabricated and tested. Note that the Poisson’s ratio of sandwich panel samples is equal to the Poisson’s ratio of inner cores (i.e., chiral cuboids), while the Poisson’s ratio of solid sample is the Poisson’s ratio of molding material (i.e., soft nylon). Fig. S7 presents the sandwich panel and solid samples used in the ball rebounding tests. The Poisson’s ratio of chiral cuboids with different $t$ under axial tension and compression are obtained from the parametric studies via numerical simulations (Results and Discussion). Defining the average of Poisson’s ratio under axial tension and compression as the effective Poisson’s ratio. Table S3 summarizes the tensile, compressive and effective Poisson’s ratio of used sandwich panel and solid samples.


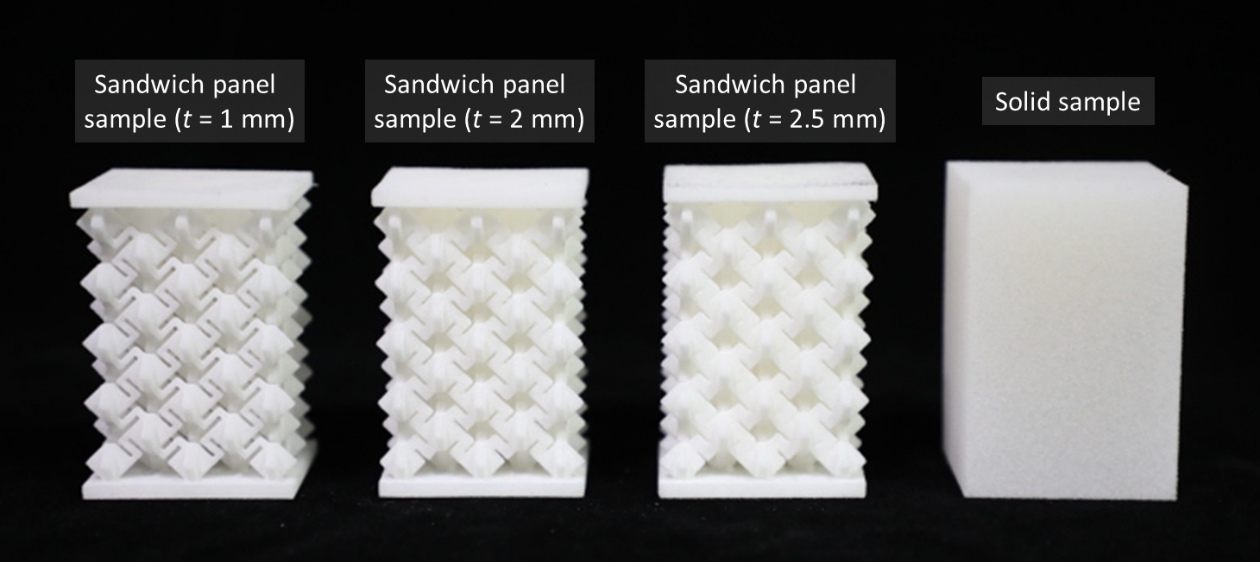


**Fig. S7.** Sandwich panel and solid samples used in the ball rebounding tests.

**Table. S3.** Tensile, compressive and effective Poisson’s ratio of sandwich panel and solid samples.

|  | Tensile Poisson’s ratio | Compressive Poisson’s ratio | Effective Poisson’s ratio |
| --- | --- | --- | --- |
| Sandwich panel sample ($t=1 \mathrm{mm}$) | -0.438 | -0.49 | -0.464 |
| Sandwich panel sample ($t=2 \mathrm{mm}$) | -0.062 | -0.092 | -0.077 |
| Sandwich panel sample ($t=2.5 \mathrm{mm}$) | 0.1 | 0.082 | 0.091 |
| Solid sample | 0.4 | 0.4 | 0.4 |

# Supplementary Note 8: Free body diagram showing the relationship between $\boldsymbol{F}$ and $\boldsymbol{F}_{\boldsymbol{1}}$

Fig. S8 presents the free body diagram (FBD) to validate the relationship between $F$ and $F_{1}$ given in Eq. (1). Specifically, when the chiral cuboids are subjected to an axial force $F$ and reach the equilibrium state, the areas 1 of Z-shaped ligaments inside the chiral cuboids will generate the responsive forces $F_{1}$. According to the principle of mechanical equilibrium, the components in the $z$ direction of responsive forces $F_{1}$ of Z-shaped ligaments in each layer is equal to the axial external force $F$. Therefore, the relationship of $F$ and $F_{1}$ can be expressed as:

| $F=NF_{1}\cos\frac{\pi}{4},$ | (S1) |
| --- | --- |

where $N$ denotes the number of Z-shaped ligaments in each layer of chiral cuboids, which can be written as:

| $N=\left( N_{x}-1 \right)N_{y}+\left( N_{y}-1 \right)N_{x}=2N_{x}N_{y}-N_{x}-N_{y}.$ | (S2) |
| --- | --- |

Substituting Eq. (S2) into Eq. (S1), we have

| $F=\frac{\sqrt{2}}{2}\left( 2N_{x}N_{y}-N_{x}-N_{y} \right)F_{1}.$ | (S3) |
| --- | --- |

Consequently, the relationship between $F$ and $F_{1}$ given in Eq. (1) is verified.


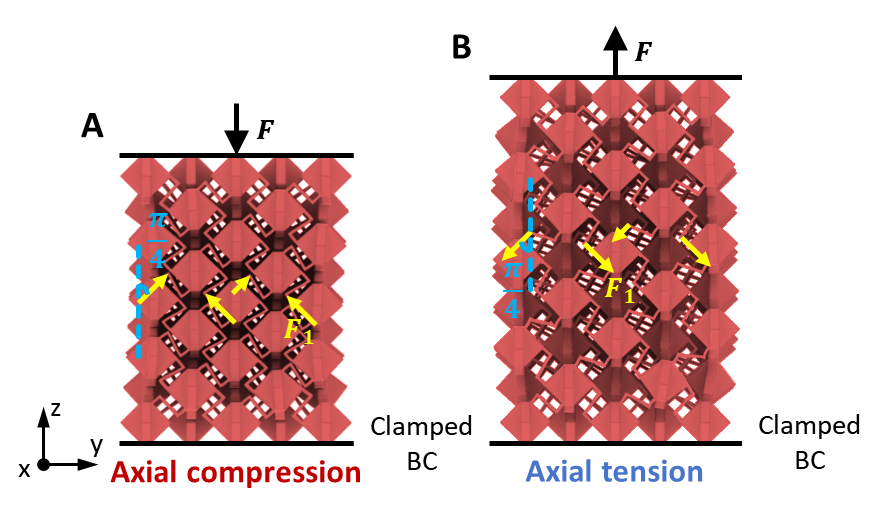


**Fig. S8.** FBD showing the relationship between $F$ and $F_{1}$ under (A) axial compression and (B) tension.

# Supplementary Note 9: Effectiveness assessment of theoretical models

This section evaluates the accuracy of developed theoretical models in Methods and conducts the error analysis. Figs. S9A and S9B compare the elastic modulus of chiral cuboids with different $t$ between theoretical analysis and numerical simulations, while Figs. S9C and S9D compare the Poisson’s ratio of chiral cuboids with different $t$ between theoretical analysis and numerical simulations. The results indicate that the theoretical models can precisely predict the elastic modulus of chiral cuboids, while unable to effectively characterize the Poisson’s ratio. Furthermore, the relative error between theoretical analysis is proportional to $t$. The causes are analyzed as follows: in theoretical analysis, the deformation of chiral cuboids under axial tension and compression originates from the bending of areas 2 of Z-shaped ligaments. However, as $t$ is increased, the bending deformation of Z-shaped ligaments is decreased, while the contributions of axial and shear deformations to the total deformation is enlarged. Consequently, the prediction accuracy of theoretical models is negatively correlated with $t$, and the impact of $t$ on the theoretical model of Poisson’s ratio is greater than the theoretical model of elastic modulus.


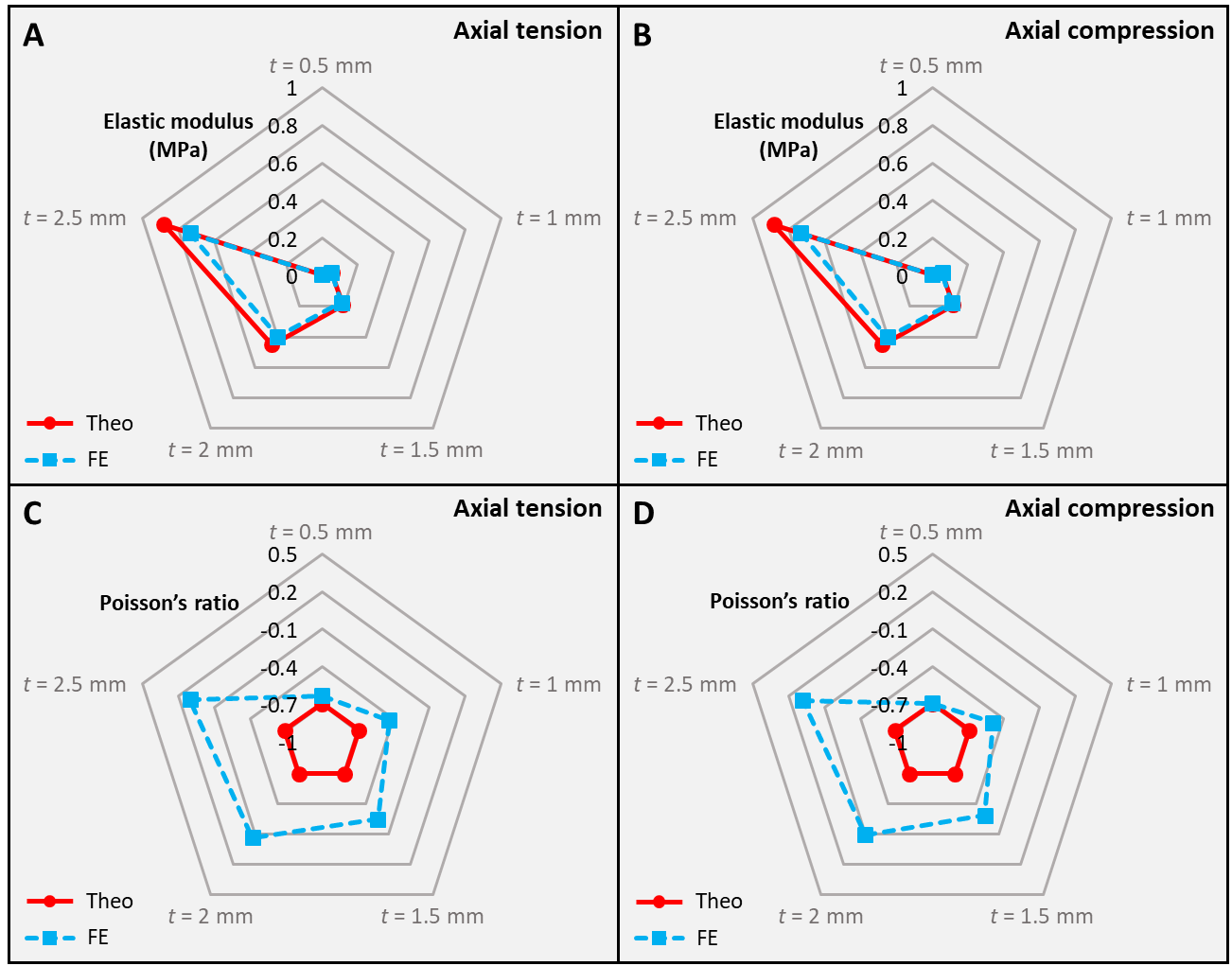


**Fig. S9.** Accuracy assessment of theoretical models via numerical simulations. Radar charts to compare the elastic modulus of chiral cuboids with different thickness of Z-shaped ligaments $t$ between theoretical analysis and numerical simulations under (A) axial tension and (B) compression. Radar charts to compare the Poisson’s ratio of chiral cuboids with different $t$ between theoretical analysis and numerical simulations under (C) axial tension and (D) compression.

**Movie S1.** Wholly auxetic response of coupling chiral cuboids in experiments.

**Movie S2.** Poisson’s ratio sign-switching of coupling chiral cuboids in numerical simulations.

**Movie S3.** Wholly auxetic response of coupling chiral cuboids in theoretical analysis.

**Movie S4.** Ball rebounding tests to validate the impact-resistant performance of sandwich panels based on coupling chiral cuboids.

1. ^1^Corresponding authors: [pjiao@zju.edu.cn](mailto:pjiao@zju.edu.cn) (P.J) and [alavi@pitt.edu](mailto:alavi@pitt.edu) (A.H.A). [↑](#footnote-ref-1)
